# Comparison of standard- and mini-percutaneous nephrolithotomy for staghorn stones

**DOI:** 10.1080/2090598X.2021.1878670

**Published:** 2021-01-21

**Authors:** Sanjay Khadgi, Ahmed R. El-Nahas, Mohamed El-Shazly, Abdullatif Al-Terki

**Affiliations:** a Department of Urology, Vayodha Hospital, Kathmandu, Nepal; b Urology and Nephrology Center, Mansoura University, Mansoura, Egypt; c Urology Unit, Al-Amiri Hospital, Kuwait City, Kuwait; d Urology Department, Menoufia University, Menoufia, Egypt

**Keywords:** Mini-PCNL, standard-PCNL, staghorn, renal calculi, percutaneous nephrolithotomy

## Abstract

**Objectives**: To compare the outcomes of standard- and mini-percutaneous nephrolithotomy (PCNL) for the treatment of staghorn stones.

**Patients and Methods**: The data of consecutive adult patients who underwent PCNL for the treatment of staghorn stones, between July 2015 and December 2019 from three hospitals, were retrospectively reviewed. All cases were performed in a prone position under fluoroscopic guidance. The nephrostomy tracts were dilatated to 30 F in standard-PCNL and to 18–20 F in mini-PCNL. Stones were fragmented with pneumatic lithotripsy in both groups. Fragments were removed with forceps in the standard-PCNL, while they were evacuated through the sheath using the vacuum clearance effect in mini-PCNL. A ureteric stent was inserted after mini-PCNL, while a nephrostomy tube was inserted after standard-PCNL.

**Results**: The study included 153 patients; 70 underwent standard-PCNL and 83 underwent mini-PCNL. The stone-free rates of PCNL monotherapy were comparable for both groups (83% for mini-PCNL and 88.6% for standard-PCNL, *P* = 0.339). The incidence (12% vs 24.3%, *P* = 0.048) and severity of complications were significantly lesser with mini-PCNL (*P* = 0.031). Standard-PCNL was associated with increased rate of blood transfusion (12.9% vs 2.4%, *P* = 0.013) and a significant decrease in haemoglobin (*P* = 0.018). Hospital stay was significantly longer for standard-PCNL than mini-PCNL (median stay of 6 vs 3 days, *P <* 0.001).

**Conclusions**: The efficacy of mini-PCNL was comparable to standard-PCNL in the treatment of staghorn stones. The advantages of mini-PCNL included a lesser incidence and severity of complications, and shorter hospital stay.

## Introduction

Staghorn stones are complex branching renal calculi that represent a special challenge for treatment. Different classifications and scores have been reported for staghorn stones [1,2]. Using the Guy’s Stone Score partial staghorn stones are classified as Score III and complete staghorn as Score IV [2]. Standard-percutaneous nephrolithotomy (PCNL) is the recommended treatment by major guidelines [3,4]. This implies a large track of 24–30 F. A considerable number of patients require multiple tracts to achieve a stone-free status [5]. Consequently, there is a high-rate of complications when using standard-PCNL for treating staghorn stones, particularly bleeding requiring blood transfusion or angiographic embolisation, which is the most dangerous complication [6]. This has been attributed to the large tract size and the need for multiple tracts [7–9].

Recently, miniaturised PCNL techniques (such as mini-, super-mini-, ultra-mini-, and micro-PCNL) have been introduced and gained acceptance as alternatives to standard-PCNL [10]. Multiple studies have reported comparable effectiveness to standard-PCNL in the treatment of small, medium sized and non-complex stone burden [11–13]. The main advantages over standard-PCNL were fewer bleeding complications, less postoperative pain, and more ability to perform tubeless PCNL [11,14].

A few studies from one hospital in China evaluated mini-PCNL for the treatment of complex or staghorn stones [15–18], one of these studies compared mini- with standard-PCNL [17]. Another study in the Chinese language was also published [19].

The present study was conducted to compare the outcomes of standard- with mini-PCNL in the treatment of staghorn stones.

## Patients and methods

All procedures performed in the study were in accordance with the ethical standards of the Institutional and National Research Committee and with the 1964 Helsinki declaration and its later amendments or comparable ethical standards. The data of patients who underwent PCNL for the treatment of staghorn stones, between July 2015 and December 2019 from three hospitals, were retrospectively reviewed. Standard-PCNL cases were done in two hospitals (in Kuwait and Egypt) and mini-PCNL cases were performed in the third hospital (in Nepal). An experienced endourologist performed the cases in each hospital. The study included consecutive adult patients (aged ≥18 years) with partial or complete staghorn stones (Guy’s Stone Score III or IV). Patients with congenital renal anomalies were excluded.

For all patients in both groups, non-contrast CT (NCCT) was performed for defining staghorn complexity and planning numbers and locations of percutaneous tracts. All patients with infected preoperative urine cultures received antibiotics according to the sensitivity test. They were scheduled for PCNL when the urine culture became sterile. Patients with negative urine cultures received prophylactic antibiotics before induction of anaesthesia. Fluoroscopic-guided percutaneous renal access was done in the prone position. When multiple tracts were deemed necessary, they were all established before dilatation of any tract.

In standard-PCNL, the tracts were dilatated to 30 F using Alken metal dilators or balloon dilatation (Ultraxx™, Cook Medical, Bloomington, IN, USA) then an Amplatz sheath was placed. A 24-F nephroscope (Richard Wolf GmbH, Knittlingen, Germany) was used. Stones were disintegrated with pneumatic lithotripsy (Swiss Lithoclast Master, EMS, Nyon, Switzerland) and fragments were extracted with forceps. At the end of the procedure, a ureteric catheter was inserted, and a 16-F nephrostomy tube was fixed for 2 days unless complications occurred.

In mini-PCNL, under spinal anaesthesia, the tracts were dilatated to 18–20 F using a single-step dilator. A 12-F nephroscope (MIP-M, Karl Storz Endoskope, Tuttlingen, Germany) was used. Stones were fragmented with pneumatic lithotripsy and fragments were evacuated through the sheath using the vacuum clearance effect and retrograde saline push through the ureteric catheter during withdrawal of the nephroscope. At the end of the procedure, a ureteric stent was inserted, and the sheath was removed under direct vision without insertion of a nephrostomy tube (tubeless), unless there were complications (such as bleeding or perforation of the calyceal system) or significant residual stones planned for second look.

The ureteric stent was removed after 1–2 weeks under local anaesthesia in both groups if there were no complications or residual stones. The operative time was measured from ureteric catheter insertion until the insertion of the nephrostomy tube in standard-PCNL or removal of the tract sheath in mini-PCNL. Intraoperative complications were recorded and 30-day postoperative complications were graded according to the modified Clavien classification [20]. The stone-free status of PCNL monotherapy was evaluated with a plain abdominal radiograph of the kidneys, ureters and bladder (KUB) for radiopaque stones and NCCT for lucent stones before hospital discharge. Stone-free status was defined as no or small calyceal residuals of ≤3 mm without infection [21].

### Statistical analysis

The data were stored and analysed using the Statistical Package for the Social Sciences (SPSS®), version 20 (IBM Corp., Armonk, NY, USA). Both groups were compared for demographics, stone burden according to the Guy’s Stone Score System, number and locations of percutaneous tracts, operation time, incidence and severity of complications, blood transfusion rate, decrease in haemoglobin level after surgery, hospital stay, and stone-free rate (SFR). Nominal data were compared using the chi-square or Fischer’s exact tests and continuous data were compared with the *t*-test or Mann–Whitney *U*-test as appropriate. Significance was set at *P* < 0.05.

## Results

The present study included 153 patients; 70 underwent standard-PCNL and 83 underwent mini-PCNL. The preoperative data are summarised in Table 1. Patients who underwent standard-PCNL were older and more obese (*P* < 0.001). Operative, postoperative and outcome data are presented in Table 2. Patients in the standard-PCNL group required multiple more tracts than mini-PCNL (*P* < 0.001). The SFRs of PCNL monotherapy were comparable for both groups (*P* = 0.339). However, the requirement of second sessions of PCNL was significantly lower in the mini-PCNL group (*P* = 0.003). Auxiliary procedures in the standard-PCNL group included: shockwave lithotripsy (SWL) in five patients, flexible ureterorenoscopy (URS) in three and semi-rigid URS in one, while two patients refused further treatment of significant residual stones. In the mini-PCNL group, SWL was performed for seven patients and semi-rigid URS for two, while five refused further treatment.

**Table 1.** Preoperative characters of mini- and standard-PCNL

Variable	Mini-PCNL 83 patients	Standard-PCNL 70 patients	*P*
Gender, *n* (%)			0.368
Male	44 (53)	32 (45.7)	
Female	39 (47)	38 (54.3)	
Side, *n* (%)			0.808
Right	36 (43.4)	21 (41.4)	
Left	47 (56.6)	41 (58.6)	
ASA Score, *n* (%)			0.807
1 2	43 (51.8) 38 (45.8)	36 (51.4) 31 (44.3)	
3	2 (2.4)	3 (4.3)	
Preoperative urine cultures, *n* (%)			0.102
Sterile Infected	58 (70) 25 (30)	40 (57) 30 (43)	
Stone recurrence, *n* (%)			0.232
First time	78 (94)	62 (88.6)	
Recurrent	5 (6)	8 (11.4)	
Hydronephrosis, *n* (%)			0.280
No	22 (26.5)	27 (38.6)	
Mild to moderate	57 (68.7)	40 (51.7)	
Marked	4 (4.8)	3 (4.3)	
Stone opacity, *n* (%)			0.379
Opaque	55 (66.3)	51 (73)	
Lucent	28 (33.7)	19 (27)	
Guy’s Stone Score, *n* (%)			0.110
III	34 (41)	20 (28.6)	
IV	49 (59)	50 (71.4)	
Age, years, mean (SD)	43.7 (13.9)	51.9 (9.7)	>0.001
BMI, kg/m^2^, mean (SD)	29 (3.3)	34 (6)	>0.001

ASA, American Society of Anesthesiologists.

**Table 2.** Operative, postoperative and outcomes of mini- and standard-PCNL

Variable	Mini-PCNL 83 patients	Standard-PCNL 70 patients	*P*
Number of tracts, *n* (%)			<0.001
1	54 (65.1)	21 (30)	
2	20 (24.1)	31 (44.3)	
3	9 (10.8)	18 (25.7)	
Skin puncture, *n* (%)			0.859
Subcostal	26 (31.3)	21 (30)	
Supracostal	57 (68.7)	49 (70)	
Calyceal puncture, *n* (%)			<0.001
Lower	6 (7.2)	10 (14.3)	
Middle	44 (53)	10 (14.3)	
Upper	4 (4.8)	1 (1.4)	
Multiple	29 (34.9)	49 (70)	
Number of PCNL sessions, *n* (%)			0.003
1 2	73 (88) 10 (12)	48 (68.6) 22 (31.4)	
Outcome, *n* (%)			0.339
Stone free	69 (83)	62 (88.6)	
Significant residuals	14 (17)	8 (11.4)	
Complications, *n* (%)			0.048
No	73 (88)	53 (75.5)	
Yes	10 (12)	17 (24.3)	
Modified Clavien Classification Grade, *n* (%)			0.031
I	6 (7.2)	3 (4.3)	
II	2 (2.4)	9 (12.9)	
IIIa	2 (2.4)	5 (7.1)	
Blood transfusion, *n* (%)	2 (2.4)	9 (12.9)	0.013
Operative time, min, mean (SD)	90 (32.4)	99.6 (32.9)	0.071
Haemoglobin deficit, g/L, median (range)	10 (1–38)	15 (1–46)	0.018
Hospital stay, days, median (range)	3 (2–5)	6 (2–10)	<0.001

The incidence and severity of complications were significantly lesser in the mini-PCNL group than standard-PCNL group (*P* = 0.048 and *P* = 0.031, respectively). Intraoperative bleeding leading to premature termination of the procedure occurred in one and five patients in the mini- and standard-PCNLs, respectively. Grade I modified Clavien complications in the mini-PCNL group included fever (<38.5°C) in five patients, which was treated with intravenous antibiotics, and perinephric urinoma in one patient that was managed conservatively. In the standard-PCNL group, postoperative fever developed in three patients. Postoperative haematuria was observed in one and four patients, respectively. There was a significant difference in the haemoglobin decrease between both groups (*P* = 0.018). The rate of blood transfusion (Grade II complications) was significantly more in the standard-PCNL group (12.9% vs 2.4%, *P* = 0.013). Grade III complications in mini-PCNL included replacement of mispositioned ureteric stent leading to urinary leakage through the nephrostomy site and unplanned removal of the ureteric stent 3 days after hospital discharge due to severe symptoms that were not tolerated by the patient. While in the standard-PCNL group, four patients required ureteric stent insertion for treatment of urinary leakage and one patient required intercostal chest tube for hydrothorax. There were no Grade IV and V complications. The hospital stay was significantly longer in the standard-PCNL group compared with the mini-PCNL group (median stay of 6 vs 3 days, *P* < 0.001).

## Discussion

Standard-PCNL is the recommended treatment for staghorn stones because of the higher SFRs when compared with minimally invasive modalities and lower complications in comparison with open surgery [3]. However, the incidence and severity of standard-PCNL complications are significantly higher than other minimally invasive modalities [20]. Mini-PCNL techniques represent a natural evolution to decrease complications of standard-PCNL. In the present study, the overall complication rate of standard-PCNL was double that of mini-PCNL (24% vs 12%, *P* = 0.048). The reason for this was the lower incidence of bleeding requiring blood transfusion with mini-PCNL (2.4% vs 12.9%, *P* = 0.013). This is logical because of the great difference in surface area of renal parenchymal violation between 18–20 F and 30 F tracts. The same observation was reported by all studies comparing standard- with mini-PCNL for treatment of renal calculi [9,14]. Another factor was the need for multiple tracts in 70% of standard-PCNLs vs 35% of mini-PCNLs. This is attributed to the feasibility of navigation of most renal calyces by a small 12-F nephroscope through a single access without damage to the calyceal necks [22]. These manoeuvres would have caused severe bleeding from calyceal neck injury if tried with large 24-F nephroscope. The increased risks of bleeding and the need for embolisation were reported with multiple standard-PCNL tracts in previous studies [6,7].

Since the introduction of mini-PCNL techniques, investigators have been exploring their potential in the treatment of different stone burdens. At the beginning, they were accepted for paediatric urolithiasis because a small tract was suitable for paediatric kidneys, but utilisation of mini-PCNL for the treatment of adult patients was resisted [23]. Then, studies of mini-PCNL showed excellent results in treatment of small renal stones of 10–20 mm [24,25]. The advantages over standard-PCNL such as fewer bleeding complications, less postoperative pain and shorter hospitalisation periods were recognised, while the main drawback was longer operative time [9,13]. Reports from Guangzhou in China showed the safety and efficacy of mini-PCNL in a huge number of patients [18,26]. Guohua *et al*. [16] reported a SFR of 93% for mini-PCNL for staghorn stones in 100 patients. While Zhao *et al*. [15] reported a SFR of 84% for two-stage multi-tract mini-PCNL for staghorn stones. These retrospective case series showed the safety and efficacy of this technique and encouraged more surgeons to perform mini-PCNL for the treatment of large, complex and staghorn stones [12,22,27].

In the present study, the SFRs were comparable (83% for mini-PCNL vs 88.6% for standard-PCNL, *P* = 0.339). The differences between both techniques were the requirement of multiple tracts and multiple sessions in standard-PCNL (*P* > 0.001 and *P* = 0.003, respectively). This is also attributed to increased ability of the surgeon to move inside the calyceal system for chasing residual fragments in mini-PCNL. Another factor was the increased clearance of stone fragments outside the surgeon’s field of vision by the vacuum cleaner effect. Zhong *et al*. [17] prospectively compared 29 mini-PCNL and 25 standard-PCNL cases for staghorn stones. The mini-PCNL technique achieved a significantly better SFR (89.7% vs 68%, *P* = 0.049) and lesser need for a second session (13.8% vs 28%, *P* = 0.048). The main difference between the Zhong et al. [17] study and the present one was the utilisation of multiple tracts in all patients in comparison with 35% in our cases of mini-PCNL.

Unlike previous studies that reported significantly longer operative time with mini-PCNL when compared to standard-PCNL [14], the results of the present study showed that mean operative time of mini-PCNL was comparable to standard-PCNL (90 vs 99.6 min, *P* = 0.071). Zhong *et al*. [17] also found a comparable median operative time for mini-PCNL and standard-PCNL for treating staghorn stones (116 vs 103 min, *P* = 0.052). Factors leading to decreased operative time in mini-PCNL in the present study include lower body mass index (BMI) and spinal anaesthesia leading to shorter time for patient’s prone positioning after fixation of the ureteric catheter. Other factors include shorter time for dilatation of a small tract and completion of the procedure through one tract in 65% of the patients. The advantage of a shorter hospitalisation time for mini-PCNL, which was reported by previous studies for non-staghorn stones [9,11,13], was also confirmed in the present study (median hospital stay of 3 vs 6 days, *P* < 0.001). This is the result of lower complications, less need for a second PCNL session, and absence of nephrostomy tube in mini-PCNL.

Limitations of the present study include the retrospective design, comparing two ethnic groups of patients with older and more obese patients in standard-PCNL and evaluation of SFR with KUB in many patients. There is still a need for a multicentre randomised controlled trial to obtain a higher level of evidence about the role of mini-PCNL in the treatment of staghorn stones. However, the superior safety profile of mini-PCNL and comparable SFRs of the present study support utilisation of mini-PCNL for the treatment of staghorn stones.

## Conclusions

The efficacy of mini-PCNL was comparable to standard-PCNL for the treatment of staghorn stone. The advantages of mini-PCNL included less overall incidence and severity of complications and shorter hospital stay.
